# Successful catheter ablation of ventricular tachycardia in a patient with congenitally corrected transposition of great arteries after double switch operation

**DOI:** 10.1186/s40064-016-1903-4

**Published:** 2016-03-16

**Authors:** Keiko Toyohara, Tomomi Nishimura, Toshio Nakanishi, Morio Shoda

**Affiliations:** Department of Pediatric Cardiology, Tokyo Women’s Medical University, Tokyo, Japan; Department of Cardiology, Tokyo Women’s Medical University, 8-1 Kawada-cho, Shinjuku-ku, Tokyo, 162-8666 Japan

**Keywords:** Catheter ablation, Ventricular tachycardia, Congenitally corrected transposition of great arteries, Double switch operation

## Abstract

Ventricular tachycardia (VT) may cause sudden death late after repair of congenital heart disease. Radiofrequency catheter ablation (CA) of VT can be effective but may be hampered by hypertrophied myocardium or prosthetic material. A 33-year-old man with congenitally corrected transposition of the great arteries (ccTGA) had undergone a double switch operation (DSO) with the combined Mustard and Rastelli procedures when he was 10 years old. He developed sustained VT 16 years after the surgery. An electrophysiological study was performed using a 3D mapping system. During the VT, abnormal fragmented potentials were identified in the right ventricular side near the patch of ventricular septal defect. Radiofrequency energy was delivered at the site with changes in the QRS morphology. Electroanatomical mapping of the left ventricle was performed via a retrograde transaortic approach. Successful ablation of the fractionated potentials was likewise achieved on the left side. We report, to our knowledge, the first case of a successful radiofrequency CA of VT in a patient with ccTGA after a DSO. A slow conduction zone, which was proved to be part of the tachycardia substrate, existed around the patch of ventricular septal defect. Fragmented activities during VT were recorded from both ventricles. The tachycardia circuit was eliminated after ablating the right and left sides of the ventricular septal defect patch.

## Background

Surgical treatment has improved the long-term prognosis of patients with congenital heart disease (CHD), but late ventricular tachycardia (VT) remains a risk for patients who have undergone ventriculotomies (Murphy et al. 1993; Norgaard et al. 1999]. Catheter ablation (CA) of VT after repair of CHD may be difficult because of unmappable VT and complex anatomy. Insights into the relationship between anatomical obstacles identified through substrate mapping techniques and critical reentry circuit isthmuses might facilitate ablation (Zeppenfeld et al. 2007). In some cases, CA of the VT around the patch of the ventricular septal defect (VSD) may be necessary on the right ventricle (RV) and the left ventricle (LV) (Kapel et al. 2014).

## Case presentation

**Fig. 1:**
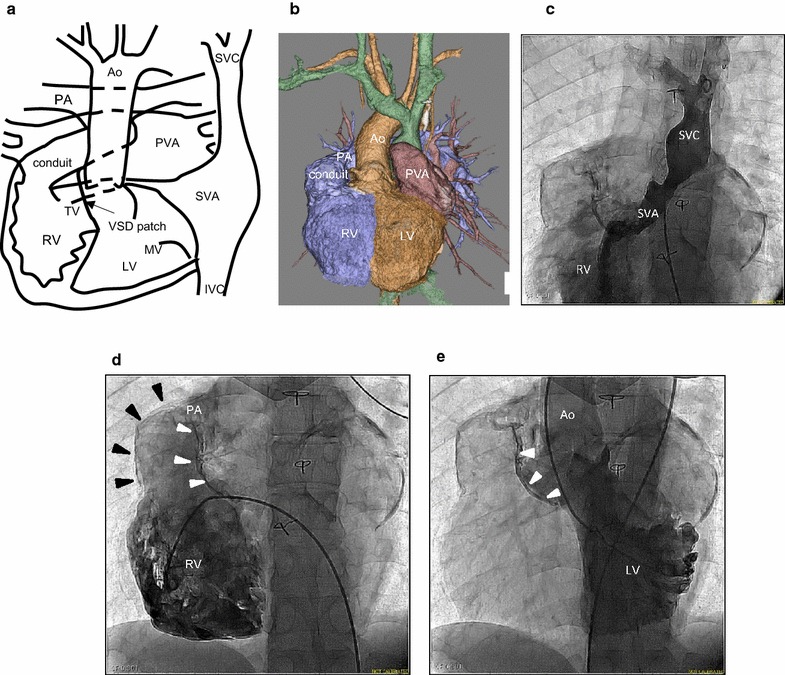
**a–e** Anatomy of a case with congenitally corrected transposition of the great arteries (ccTGA) following a double switch operation (DSO): Mustard + Rastelli. Schema of ccTGA following DSO and VSD closure. Mustard route: left SVC and IVC to SVA, and Rastelli route: RV to pulmonary artery PA via conduit (**a**), three-dimensional computed tomographic (3DCT) view of the great arteries, cardiac chambers, and Rastelli conduit (**b**), angiography of the left SVC (**c**), angiography of the RV, showing calcification of the Rastelli conduit (*black triangles*) and calcification of the VSD patch (*white triangles*) (**d**), angiography of the LV showing calcification of the VSD patch (*white triangles*) (**e**). * *Ao* aorta, *IVC* inferior vena cava, *LV* left ventricle, *MV* mitral valve, *PA* pulmonary artery, *PVA* pulmonary venous atrium, *RV* right ventricle, *SVA* systemic venous atrium, *SVC* superior vena cava, *TV* tricuspid valve, *VSD* ventricular septal defect

A 33-year-old man with congenitally corrected transposition of the great arteries (ccTGA) had situs inversus of the viscera and atria, D-loop of the ventricles, and d-transposition of the great arteries: {I, D, D}, VSD and pulmonary stenosis. He had undergone a double switch operation (DSO) with the combined Mustard and Rastelli procedures (Fig. 1a) when he was 10 years old. The operation involved patch closure of the VSD and transection of the main pulmonary artery (PA), with the PA stump doubly oversewn and ligated. The distal end of the tricuspid conduit was anastomosed to the distal PA. Three-dimensional computed tomography (3DCT) and angiography showed the Mustard route (left-sided superior vena cava and inferior vena cava to systemic venous atrium with calcified wall) and the Rastelli route (RV to PA via the calcified conduit) (Fig. 1b, c). The VSD patch between the RV conduit and the LV was prominently calcified (Fig. 1d, e). He developed sustained VT, which required cardioversion 16 years after the surgery, and he was admitted for CA of the VT.

**Fig. 2:**
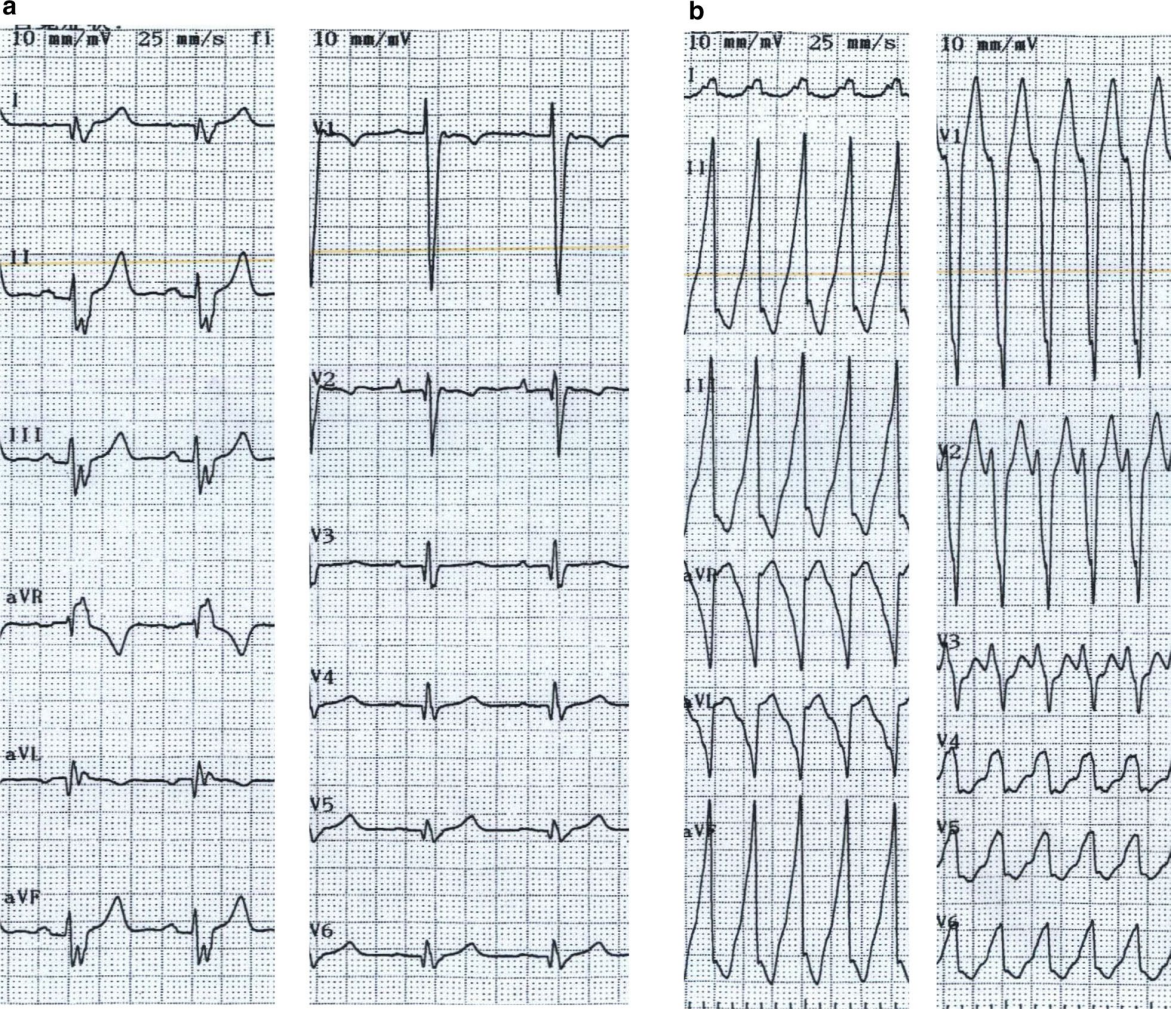
Twelve-lead ECG recorded during sinus rhythm (**a**), twelve-lead ECG recorded during ventricular tachycardia (**b**)

**Fig. 3:**
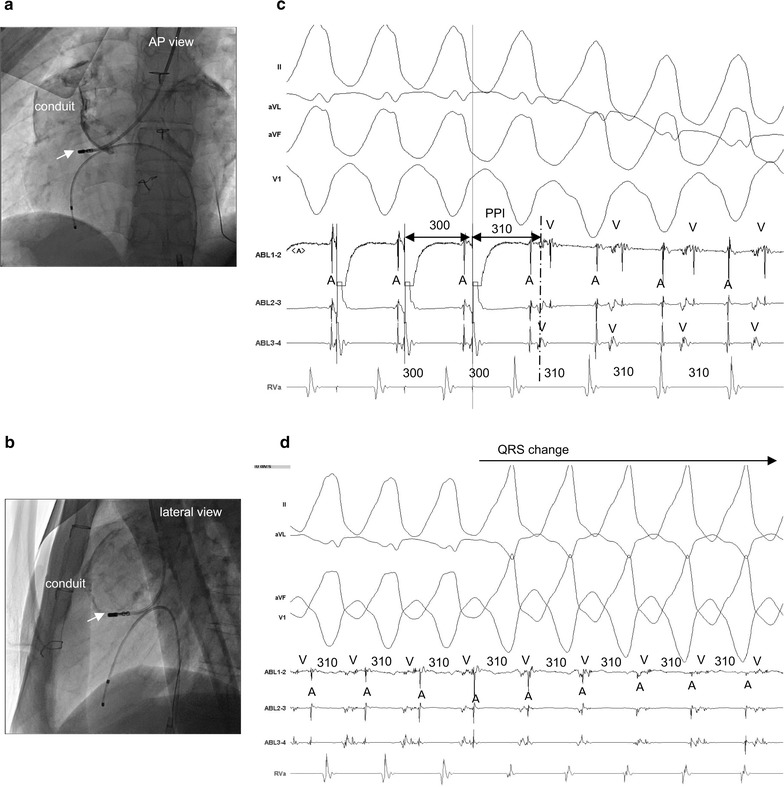
**a–d** Catheter ablation with fractionated diastolic potentials near VSD patch from the right ventricle. Ablation site (*white arrow*) by an ablation catheter via the left jugular vein [anteroposterior (AP) view (**a**), lateral view (**b**)], **c**oncealed entrainment is achieved and post-pacing interval (PPI) coincides with the cycle length of VT (310 ms) (**c**), change in the QRS morphology of VT during catheter ablation. * *VSD* ventricular septal defect, *VT* ventricular tachycardia

**Fig. 4:**
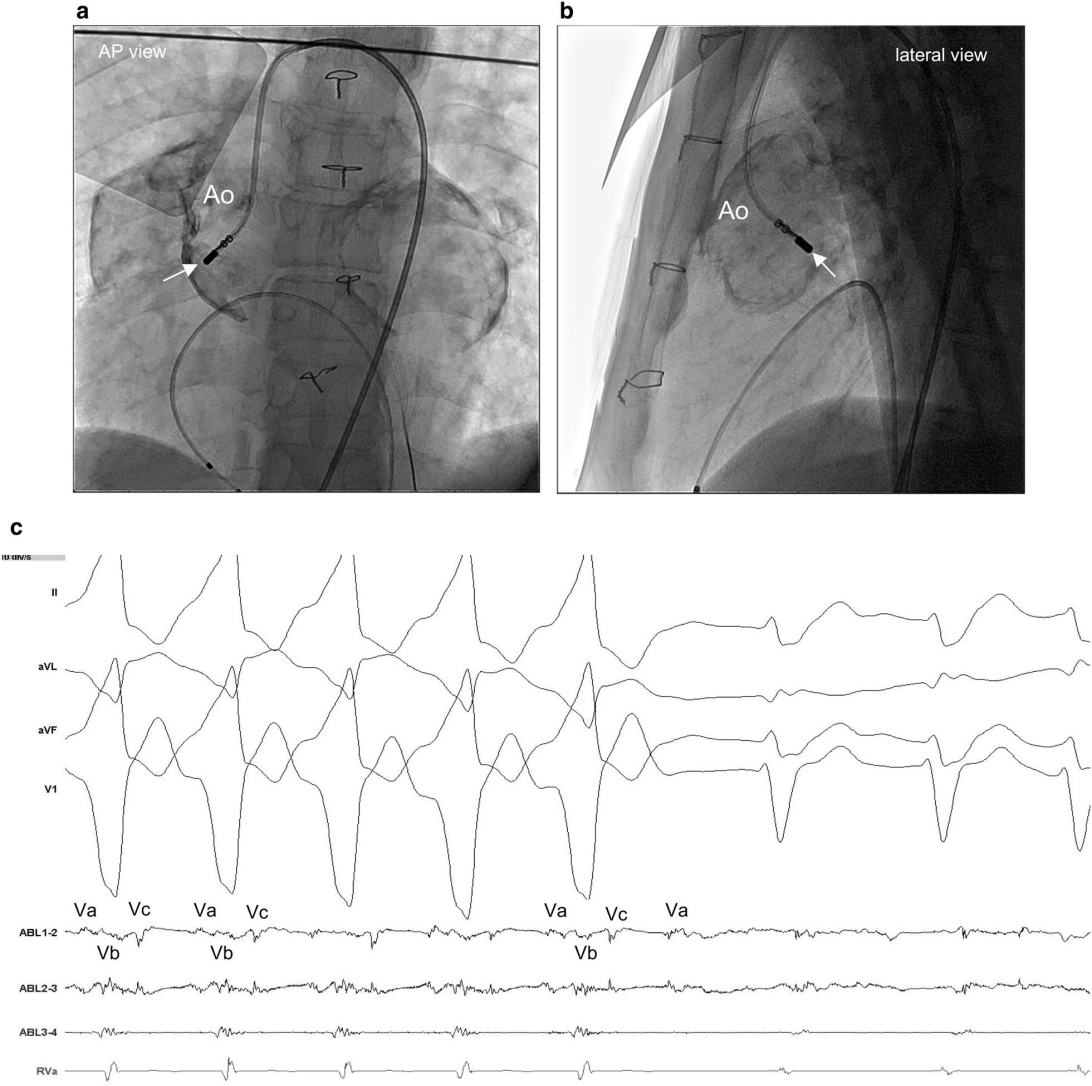
**a–c** Catheter ablation near VSD patch from the left ventricle. Ablation site (*white arrow*) with fractionated diastolic potentials (Va, Vb, Vc) near the VSD patch from the left ventricle by an ablation catheter via the aorta [anteroposterior (AP) view (**a**), lateral view (**b**)], VT is terminated during catheter ablation by a conduction block between Va and Vb (**c**). * *VSD* ventricular septal defect, *VT* ventricular tachycardia

**Fig. 5:**
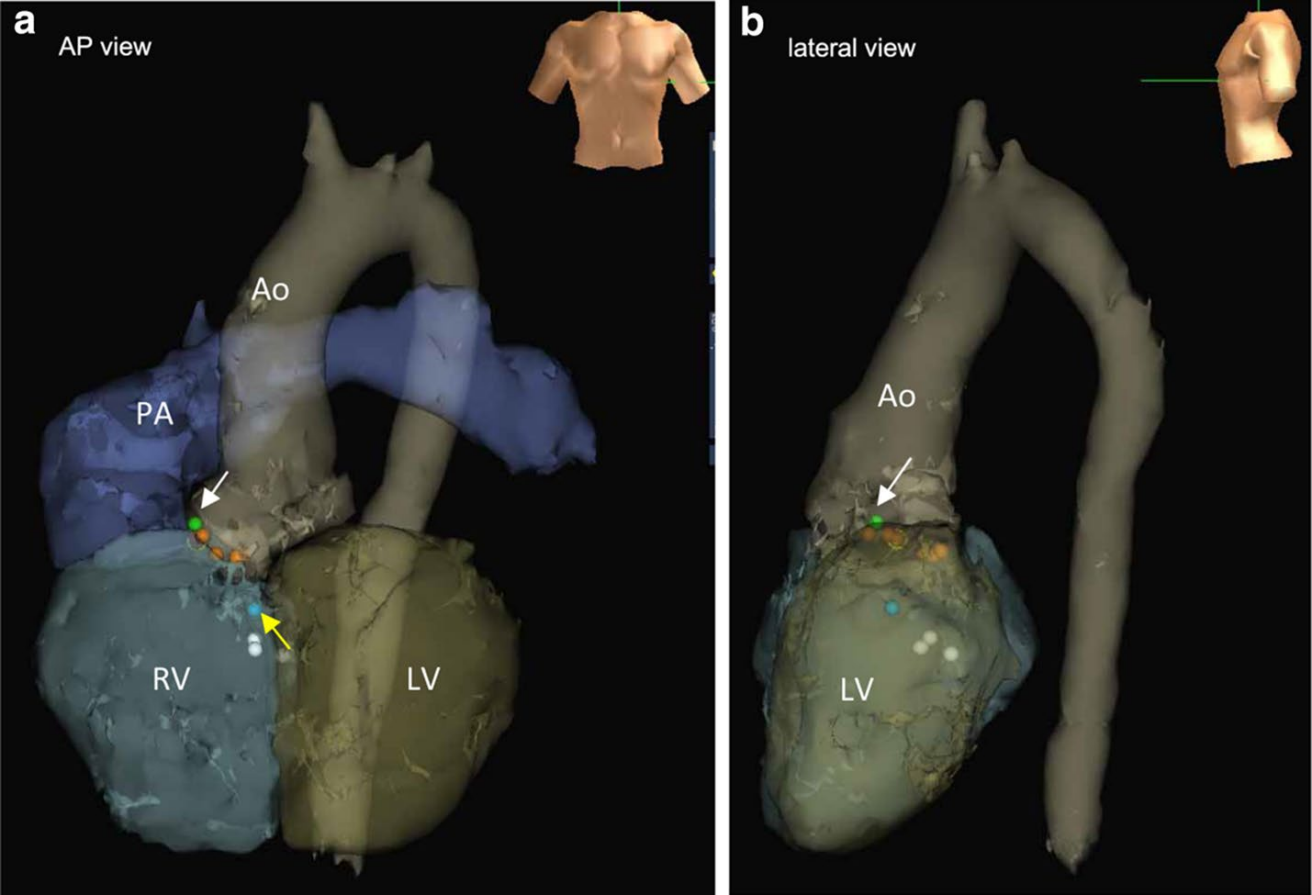
**a, b** The sites of ablation on the 3DCT of the heart integrated in the Ensite platform [anteroposterior (AP) view (**a**), left lateral view (**b**)]. A *blue tag* with a *yellow arrow* shows the first ablation site from RV where QRS morphology of VT changed during radiofrequency application. A *green tag* with a *white arrow* shows the second ablation site from LV where VT was terminated. * *Ao* aorta, *LV* left ventricle, *PA* pulmonary artery, *RV* right ventricle

An electrophysiological study (EPS) was performed under local anesthesia. At baseline, the heart showed sinus rhythm (Fig. 2a). A 5-French bipolar catheter was positioned in the RV apex. The clinical VT was reproducibly induced by programmed electrical stimulation at the RV apex with one extrastimulation. The VT was with left bundle branch block with an inferior axis QRS morphology (Fig. 2b). The tachycardia was stable and hemodynamically well-tolerated. No VT with any other morphology was inducible. Although we could not approach the RV conduit via the inferior vena cava, we could easily reach the RV conduit via the left jugular vein by using an irrigation catheter (Coolflex^®^, St. Jude Medical, St. Paul, MN, USA) (Fig. 3a, b), by using a 3D mapping system (Ensite-NavX™ system, St. Jude Medical, St. Paul, MN, USA). During the clinical VT, abnormal fragmented potentials were not identified in the conduit. While drawing the mapping catheter from the conduit to the tricuspid valve at the anterior side near the VSD patch, fractionated diastolic potentials were recorded (Fig. 3c). At this site, the concealed entrainment was achieved, and the post-pacing interval coincided with the VT cycle length (Fig. 3c). Radiofrequency energy with a target temperature of less than 40 °C and the power output titrated up to 40 W was delivered at the site of the fractionated diastolic potentials from the RV side, with changes in the QRS morphology without prolongation of the VT cycle length (Fig. 3d). Thus, mapping of the LV was necessary, and was performed via a retrograde transaortic approach (Fig. 4a, b). Successful ablation of the fractionated potentials was likewise achieved on the left side (Fig. 4c). The sites of ablation are shown on the 3DCT of the heart integrated in the Ensite platform (Fig. 5a, b). The VT was terminated by the application of radiofrequency energy, and it became non-inducible. The patient remained symptom-free, and no VT episodes recurred during one-year follow-up after the CA.

## Discussion

Although surgical treatment has improved the prognosis of complex CHD, the problem of remote ventricular arrhythmias remains unresolved. For instance, the incidence of VT is 11.9 %, with an 8.3 % risk of sudden death by 35 years of follow-up after repair of tetralogy of fallot (TOF). Thus, VT is a possible cause of late sudden death (Gatzoulis et al. 2000).

Congenitally corrected TGA is characterized by discordant atrioventricular (AV) and ventriculo-arterial connections (Van Praagh 1970). Surgical approaches for ccTGA include physiological or anatomical repair. In patients undergoing the physiological procedure, the morphologic RV continues to support the systemic circulation, with disappointing late results. DSO involves an anatomical repair, wherein the LV is connected to the aorta with an arterial switch or a Rastelli procedure, and the AV concordance is established through an atrial switch procedure (Imai 1997). Although DSO remains a challenging procedure, DSO was often applied as much as possible to the patients with ccTGA to get better long-term outcomes (Shin’oka et al. 2007). The atrial switch procedure, which contains complex incisional suture lines, might promote atrial flutter and intra-atrial macroreentrant tachycardia. Due to the Rastelli procedure involving right ventriculotomy and VSD patch closure, this case developed arrhythmogenic substrates of VT. This necessitated understanding the precise anatomy of this patient, the type of ablation catheter to use and the approach to reach the target sites. The superior approach via the jugular veins is useful for mapping of the ventricles with complex anatomies. Nevertheless, manual manipulation of the ablation catheter may be difficult even by skillful and experienced hands. Magnetic robotic navigation is an alternative maneuver for complex CHD. (Ernst 2012).

In this case, the substrate of VT and the slow conduction zone existed around the suture of the VSD on the RV and the LV sides. Similar observations have been reported in VT associated with TOF, for which both the RV and LV ablations are required (Kapel et al. 2014).

After CA of the RV side, the QRS morphology of VT changed, probably due to modification of the exit from the slow conduction zone. Although we could not complete the whole activation map of the VT due to the difficulty of catheter manipulation, we were successfully able to ablate the sites of diastolic fractionated potentials.

To the best of our knowledge, there has never been a case report describing CA of VT after DSO.

## Conclusion

This report highlights ccTGA, which is of special interest because this is the first case of a successful CA of VT in a patient after a DSO and the tachycardia circuit was eliminated after ablating the right and left sides of the VSD patch.

## Abbreviations

CHD: congenital heart disease
VT: ventricular tachycardia
CA: catheter ablation
VSD: ventricular septal defect
RV: right ventricle
LV: left ventricle
ccTGA: congenitally corrected transposition of the great arteries
DSO: double switch operation
PA: pulmonary artery
3DCT: three-dimensional computed tomography
EPS: electrophysiological study
TOF: tetralogy of fallot
AV: atrioventricular
